# ADP Protects Cardiac Mitochondria under Severe Oxidative Stress

**DOI:** 10.1371/journal.pone.0083214

**Published:** 2013-12-13

**Authors:** Niina Sokolova, Shi Pan, Sarah Provazza, Gisela Beutner, Marko Vendelin, Rikke Birkedal, Shey-Shing Sheu

**Affiliations:** 1 Institute of Cybernetics, Tallinn University of Technology, Tallinn, Estonia; 2 Department of Pharmacology and Physiology, University of Rochester, Rochester, New York, United States of America; 3 Center for Translational Medicine, Thomas Jefferson University, Philadelphia, Pennsylvania, United States of America; Duke University Medical Center, United States of America

## Abstract

ADP is not only a key substrate for ATP generation, but also a potent inhibitor of mitochondrial permeability transition pore (mPTP). In this study, we assessed how oxidative stress affects the potency of ADP as an mPTP inhibitor and whether its reduction of reactive oxygen species (ROS) production might be involved. We determined quantitatively the effects of ADP on mitochondrial Ca^2+^ retention capacity (CRC) until the induction of mPTP in normal and stressed isolated cardiac mitochondria. We used two models of chronic oxidative stress (old and diabetic mice) and two models of acute oxidative stress (ischemia reperfusion (IR) and tert-butyl hydroperoxide (t-BH)). In control mitochondria, the CRC was 344 ± 32 nmol/mg protein. 500 μmol/L ADP increased CRC to 774 ± 65 nmol/mg protein. This effect of ADP seemed to relate to its concentration as 50 μmol/L had a significantly smaller effect. Also, oligomycin, which inhibits the conversion of ADP to ATP by F_0_F_1_ATPase, significantly increased the effect of 50 μmol/L ADP. Chronic oxidative stress did not affect CRC or the effect of 500 μmol/L ADP. After IR or t-BH exposure, CRC was drastically reduced to 1 ± 0.2 and 32 ± 4 nmol/mg protein, respectively. Surprisingly, ADP increased the CRC to 447 ± 105 and 514 ± 103 nmol/mg protein in IR and t-BH, respectively. Thus, it increased CRC by the same amount as in control. In control mitochondria, ADP decreased both substrate and Ca^2+^-induced increase of ROS. However, in t-BH mitochondria the effect of ADP on ROS was relatively small. We conclude that ADP potently restores CRC capacity in severely stressed mitochondria. This effect is most likely not related to a reduction in ROS production. As the effect of ADP relates to its concentration, increased ADP as occurs in the pathophysiological situation may protect mitochondrial integrity and function.

## Introduction

Ca^2+^ and ADP are the two major regulators of mitochondrial energy metabolism that function in coordination to keep the balance between the energy demand and supply. In cardiac muscle cells, during the excitation-contraction coupling, Ca^2+^ enters mitochondria to stimulate Krebs’ cycle. As such, the nicotinamide adenine dinucleotide redox potential and ATP synthesis required for cardiac workload are maintained [1]. Concomitantly, ADP generated by ATPases and kinases enters the mitochondrial matrix via the adenine nucleotide translocase (ANT) and stimulates ATP-production by F_1_F_0_-ATPase [2,3]. Therefore, both Ca^2+^ and ADP have a positive impact on ATP generation under physiological conditions. 

Ca^2+^ and ADP are also major modulators of mPTP [4–7]. But here, they function oppositely. Physiologically, the mPTP may open briefly, functioning as a mitochondrial Ca^2+^-release channel [8]. Pathologically, mitochondrial Ca^2+^-overload triggers irreversible opening of mPTP, which is a major cause of cell death. ADP, on the contrary, is a potent inhibitor of mPTP [6,7].

The molecular identity of mPTP is still unsolved. Two hypotheses exist regarding the pore-forming component. Both involve cyclophilin D (CypD) and ADP as regulators. CypD is a peptidyl-prolyl cis-trans isomerase, which binds to several proteins including ANT, the mitochondrial phosphate carrier (mPiC) and F_1_F_0_ ATPase, and increases mPTP Ca^2+^-sensitivity [9]. Irrespective of its exact site of action, it was shown that cyclosporine A (CsA) binding to CypD inhibits mPTP opening by unmasking an inhibitory P_i_-binding site [10]. Some suggest that mPiC is the pore-forming component and mainly regulated by CypD and ANT [11]. ANT in the “c” (cytosol) or “m” (matrix) conformation increases or decreases mPTP Ca^2+^-sensitivity, respectively. ADP decreases Ca^2+^-sensitivity, because its binding shifts ANT to the “m” conformation [12]. Others suggest that dimers of F_1_F_0_-ATPase are responsible for the formation of mPTP [13]. CypD also binds and inhibits F_1_F_0_-ATPase activity [14], and ADP is a potent inhibitor of the channel activity of F_0_F_1_-ATPase dimers [13].

 Until today, little is known about the effect of ADP on mPTP in diseased mitochondria, which experience increased oxidative stress, Ca^2+^-load, and energy deficiency. ADP-binding to ANT is reduced by oxidative stress [15], which might reduce the inhibiting effect of ADP on mPTP. In this paper, we wanted to address the potency of ADP as an mPTP inhibitor in diseased mitochondria with the hope to obtain clues about its mechanism of action. As noted above, ADP may exert its function by binding to either ANT or the F_0_F_1_-ATPase. But ADP may also enhance Ca^2+^-sequestration in the form of Ca^2+^-phosphate precipitates [16,17]. Furthermore, it may be speculated that part of the ADP-effect on Ca^2+^-uptake capacity is due to its reduction of ROS production [18]. Indeed, as the substrate of F_1_F_0_-ATPase, which uses the electrochemical energy stored in the proton gradient to produce ATP, ADP should reduce ROS production.

In this study, we assessed at the level of isolated mitochondria from mouse hearts how chronic and acute oxidative stress affects the effect of ADP on CRC and ROS production. As models of long-term oxidative stress, we used old mice and diabetic mice. As models of acute oxidative stress, we used IR and exposure to a low dose of t-BH. 

## Methods

### Ethics Statement

All procedures were in accordance with the NIH Guide for the Care and Use of Laboratory Animals and were approved by an Institutional Animal Care and Use Committee (University Committee on Animal Resources (UCAR) protocol 2010–030).

### Animals and models of disease and oxidative stress

Control mice: 6-8 weeks old male C57BL6 mice (n=64). Aging: 12-15-month old male C57BL6 mice (n=8). Diabetes: To induce type I diabetes, 5 weeks old male C57BL6 mice (n=12) were injected intraperitoneally with 150 mg/kg streptozotocin dissolved in 0.1 mol/L sodium citrate buffer, pH 4, prepared within 5 min of administration. Mice were given drinking water supplemented with 7.5 % sucrose for 2.5 days to avoid severe hyperglycemia. After 5 weeks the mice were diabetic and used for experiments. Exposure to t-BH: Mitochondria from 6-8 weeks old C57BL6 male mice (n=44) were exposed to 5 µmol/L t-BH for 10 min before recording Ca^2+^-uptake or ROS production. Ischemia-reperfusion injury: Male C57BL6 mice, 6-8 weeks old (n=20) were anesthetized with freshly prepared Avertin (2,2,2-tribromoethanol, 0.5 mg/kg injected intraperitoneally). Isolated hearts were retrogradely perfused in Langendorff mode under constant flow (4 ml/min) with Krebs–Henseleit buffer as in [19]. After 10 min of equilibration, hearts were subjected to 15 min of global ischemia followed by 60 min of reperfusion. 

Unless otherwise stated, the mice were euthanized by CO_2_ inhalation and sacrificed by cervical dislocation.

### Isolation of heart mitochondria

Mitochondria from 2-4 mouse hearts were isolated using the protocol of Rehncrona et al with modifications [20]. The minced heart tissue was subjected to protease treatment: it was incubated with 5 mg nagarse dissolved in 10 ml medium A for 8 minutes at room temperature while gently stirring. The protease reaction was stopped by adding 1 ml of 0.2 mg/ml of bovine serum albumin dissolved in medium A. The tissue was then homogenized with a Potter-Elvehjem homogenizer, and mitochondria were isolated by differential centrifugation. The final mitochondrial pellet was suspended in isolation medium B. 

For mitochondrial ROS generation measurements, mitochondria were Ca^2+^ depleted to minimize possible signalling of Ca^2+^ on ROS generation [21]. This procedure consisted of 15 min incubation at room temperature in Ca^2+^-depletion buffer. The mitochondria were subsequently washed several times in a Ca^2+^-depletion buffer without NaCl, EGTA and succinate. The isolated mitochondria were kept on ice and used within 4 hours. Protein concentration was determined by the Lowry method using BSA as a standard.

### Ca^2+^ uptake measurements with arsenazo III

Ca^2+^ uptake was measured with arsenazo III at room temperature as a difference in absorbance at 662 nm and the background at 692 nm using Beckman Coulter DU 800 UV-Vis spectrophotometer (Beckman Coulter Inc., Brae, CA). Isolated mitochondria (~1 mg/ml of mitochondrial protein) were added to 1 ml Ca^2+^-uptake buffer. The absorbance change upon Ca^2+^ addition was determined every 15 sec and followed for 30-70 min. Varying amounts of free Ca^2+^ were added every 2 minutes. Free Ca^2+^ concentrations were calculated using the MaxChelator program (http://www.stanford.edu/~cpatton/maxc.html).

### Measurement of mitochondrial ROS production

Mitochondrial superoxide production was determined indirectly by coupling the dismutation of superoxide to H_2_O_2_. H_2_O_2_ was detected fluorimetrically using Amplex red (10-acetyl-3,7-dihydroxyphenoxazine), which reacts with H_2_O_2_ in a 1:1 stochiometry in the presence of horseradish peroxidase (HRP), producing highly fluorescent resorufin. For these experiments, mitochondria (~0.5 mg/ml of mitochondrial protein) were added to 2 ml ROS buffer. Fluorescence was recorded at room temperature using a Cary Eclipse fluorescence spectrophotometer (Varian Inc., Walnut Creek, CA). The excitation wavelength was 563 nm and the emitted fluorescence was detected at 587 nm. A calibration signal was generated with known amounts of H_2_O_2_ at the end of each experiment.

### Measurement of mitochondrial oxygen consumption

Mitochondrial oxygen consumption was measured at room temperature using a Clark-type oxygen electrode from Hansatech (PP Systems, Boston MA).The measurements were carried out in 1 ml of respiration medium. The basal rate of respiration (State 2) was initiated by the addition of 5 mmol/L glutamate and 5 mmol/L malate as substrates. Maximal respiration rate (State 3) was measured in the presence of 1 mmol/L ADP. Respiration rates were expressed as nmol O_2_ min^-1^ mg mitochondrial protein^-1^. The respiratory control index (RCI) was calculated as RCI = State3/State2. At the end of each experiment, 8 μmol/L cytochrome c and 30 μmol/L atractyloside were added to test the intactness of the outer and inner mitochondrial membrane, respectively.

### Solutions

Krebs–Henseleit buffer for Langendorff perfusion and IR (in mmol/L): 118 NaCl, 4.7 KCl, 1.2 MgSO_4_ , 24 NaHCO_3_ , 1.2 KH_2_PO_4_, 2.5 CaCl_2_ , 11 D-glucose.

For isolation of mitochondria, medium A contained (in mmol/L): 225 mannitol, 70 sucrose, 1 EGTA and 10 HEPES, pH 7.2. Medium B contained (in mmol/L): 225 mannitol, 70 sucrose, and 10 HEPES, pH 7.2. 

Ca2+-depletion buffer contained (in mmol/L): 195 mannitol, 25 sucrose, 40 HEPES, 10 NaCl, 1 EGTA, 5 succinate, pH 7.2

For recording mitochondrial CRC, the Ca^2+^-uptake buffer contained (in mmol/L): 120 KCl, 70 mannitol, 25 sucrose, 5 KH_2_PO_4_, 0.5 EGTA, 10 HEPES, pH 7.2 in the presence of 5 mmol/L malate and 5 mmol/L glutamate as substrates and 100 μmol/L arsenazo III.

For recording ROS production, the ROS buffer contained (in mmol/L): 120 KCl, 70 mannitol, 25 sucrose, 5 KH_2_PO_4_, 0.5 EGTA, 10 HEPES, pH 7.2, 10 μmol/L Amplex® red, 1 U/ml type II HRP, and 80 U/ml Cu/Zn superoxide dismutase.

For respiration experiments, respiration medium contained (in mmol/L): 120 KCl, 70 mannitol, 25 sucrose, 5 KH_2_PO_4_, 3 MgCl_2_, 0.5 EGTA, 20 HEPES, pH 7.2.

### Statistical analysis

All values are expressed as mean ± SEM. Data were analysed by a nonparametric Mann–Whitney U test. Differences were considered significant at *P* < 0.05.

## Results

### RCI of mitochondrial preparations

The quality of the mitochondrial preparations was controlled by recording their respiration rate in the presence of substrates alone, State 2, and after addition of 1 mmol/L ADP, State 3. RCI was calculated as State 3/State 2. These values are shown in Table 1. RCI in control mice was 7.6 ± 0.2, n=10. The respiratory parameters were not different in diabetic mice. Old mice had a lower State 2 (8.6 ± 0.8, P = 0.057, n=7) and State 3 (61.3 ± 6.7, P = 0.028, n=7), but RCI was the same as in control (7.0 ± 0.4, P = 0.222, n=7). This may be attributed to a fraction of the mitochondria having already undergone mPTP. However, their CRC was the same as in control (see below). The low concentration of t-BH (5 µmol/L for 10 min) does not affect glutamate and malate-dependent respiration [22], and this was confirmed in our recordings, where State 2 and 3 and RCI was not significantly different from control. RCI was significantly lower after IR (5.3 ± 0.6, P = 0.009, n=4), consistent with an inhibition of electron transport chain activities after severe stress. 

**Table 1:** Respiration of isolated mouse heart mitochondria from control mice and different models of chronic and acute oxidative stress.

	**n**	**State 2**	**State 3**	**RCI**
**Control**	10	13.1 ± 1.4	100.8 ± 12.0	7.6 ± 0.2
**Aging**	7	8.6 ± 0.8	61.3 ± 6.7*	7.0 ± 0.4
**Diabetes**	3	14.1 ± 2.8	83.8 ± 11.3	6.2 ± 0.9
**IR**	4	9.3 ± 1.1	48.8 ± 6.4	5.3 ± 0.6**
**t-BH**	3	9.3 ± 1.4	64.1 ± 8.6	6.9 ± 0.4

Basal respiration rate in the absence of ADP (State 2), respiration rate in the presence of 1mmol/L ADP (State 3), and respiration control index (RCI = State 3/State 2). Respiration rates are expressed as nmol O_2_ min^-1^ mg mitochondrial protein**^*-*^** ^1^. Number of experiments is given in column n. Results were compared by a nonparametric Mann–Whitney U test. * and ** denote P < 0.05 and P < 0.01, respectively, compared to control.

### The protective effect of ADP is specific to [ADP]

**Figure 1:**
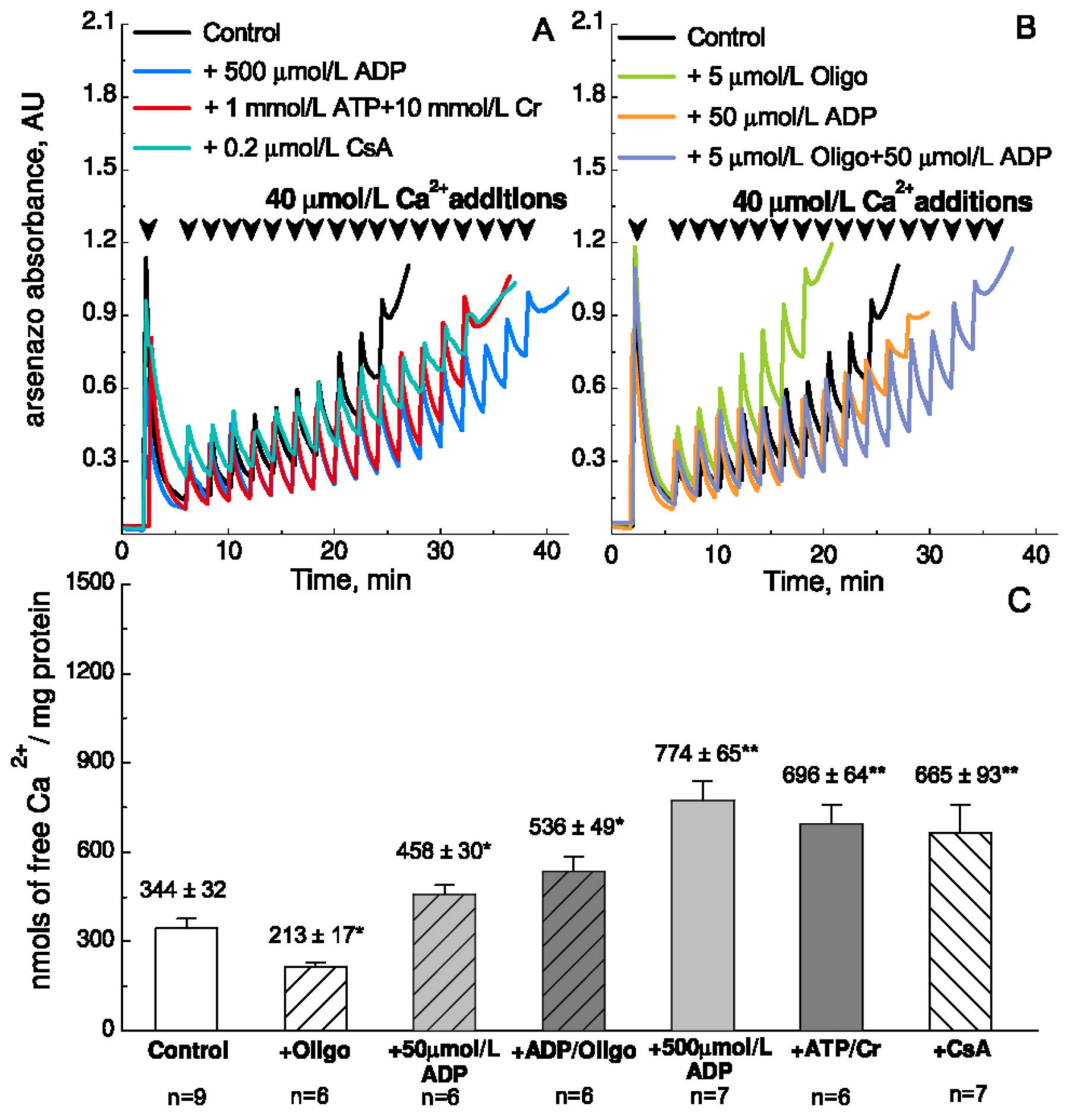
Ca^2+^-uptake capacity of isolated heart mitochondria from control mice. A-B. Representative raw traces of the Ca^2+^-uptake experiments. Mitochondria were incubated with Arsenazo III to follow extramitochondrial Ca^2+^ spectrophotometrically. Consecutive pulses leading to an increase of 40 μmol/L free Ca^2+^ were added as indicated by arrow heads. The CRC was defined as the concentration at which the mitochondria failed to accumulate more Ca^2+^ and mPTP opened to release all Ca^2+^ so far accumulated by the mitochondria. A. Traces are shown with mitochondria from control mice, 6-8 weeks old, under control conditions (no additions; black), in the presence of 500 µmol/L ADP (blue), 1 mmol/L ATP and 10 mmol/L creatine (Cr) (red), and 0.2 µmol/L cyclosporine A (CsA) (turquoise). B. Representative raw traces of the Ca^2+^-uptake experiments in the presence of 5 µmol/L oligomycin (green), 50 µmol/L ADP (orange), 5 µmol/L oligomycin and 50 µmol/L ADP (violet). C. Column diagrams of the averaged results under the conditions indicated below. The amount of Ca^2+^ was normalized to the mitochondrial content (mg protein). All values are mean ± SEM. * and ** denote significant difference P < 0.05, and P<0.01, respectively, between treatment and control. The number of experiments is indicated below.

The inhibition of mPTP by ADP has been widely reported including the seminal studies by Haworth and Hunter [23–25], in which the mPTP phenomenon was discovered. When adding ADP to the solution with isolated mitochondria in the presence of substrates, the majority will be converted into ATP. After a short period of time, an equilibrium between [ADP] and [ATP] will be reached. Oligomycin inhibits the conversion of ADP into ATP by F_1_F_0_-ATPase. Thus, [ADP] will be higher in the presence of oligomycin. We addressed the question whether the total amount of adenine nucleotides ([ADP] + [ATP]) or [ADP] specifically is important for the inhibition of mPTP. Figures 1A and B show representative traces of Ca^2+^-uptake recordings. Figure 1C summarizes the mitochondrial Ca^2+^-uptake capacity under various conditions as indicated below each column. Ca^2+^-uptake capacity for control and 500 μmol/L ADP was 344 ± 32 nmol/mg protein and 774 ± 65 nmol/mg protein, respectively, confirming that ADP inhibited mPTP potently. 1 mmol/L ATP and 10 mmol/L creatine, which stimulate mitochondrial creatine kinase to generate ADP, had a similar effect as 500 μmol/L ADP on Ca^2+^-uptake capacity (P = 0.432). A smaller dose of ADP, 50 μmol/L, had a significantly smaller effect increasing CRC to only 458 ± 30 nmol/mg protein (Figure 1C) (P = 0.003 compared to 500 μmol/L ADP; P = 0.013 compared to 1 mmol/L ATP and creatine). Inhibition of the F_1_F_0_-ATPase with 5 μmol/L oligomycin had a negative effect on Ca^2+^-uptake capacity, which decreased to approximately 62% of control (P = 0.016, Figure 1C). However, in the presence of oligomycin, 50 µmol/L ADP had a significantly larger effect on CRC, which increased from 213 ± 17 nmol/mg protein to 536 ± 49 nmol/mg protein. Thus, [ADP] is the most important inhibitor of mPTP.

### ADP recovers mitochondrial Ca^2+^-uptake capacity after acute oxidative stress

**Figure 2:**
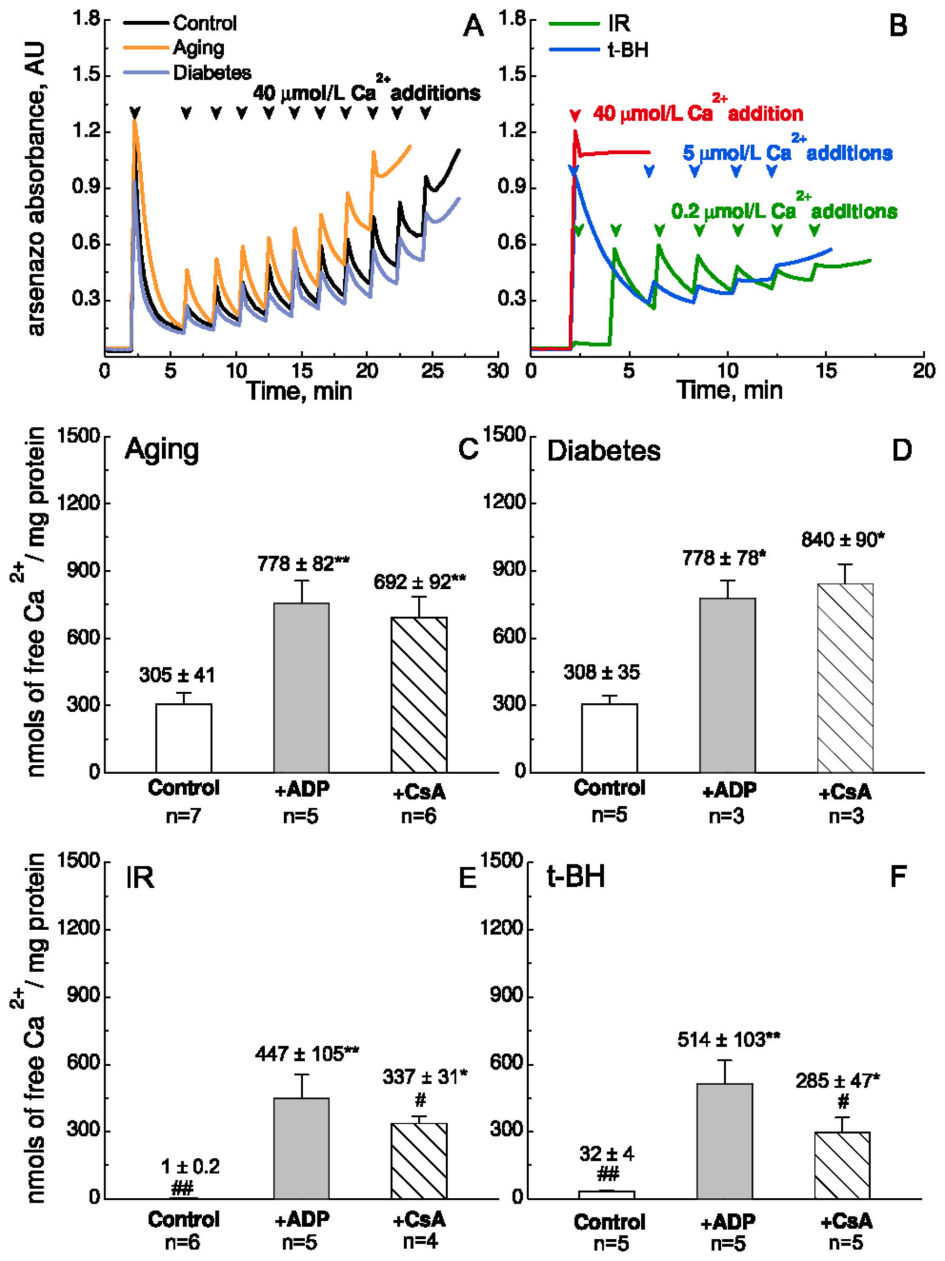
Ca^2+^-uptake capacity of isolated mouse heart mitochondria from different models of chronic and acute oxidative stress. A-B: Representative raw traces of the Ca^2+^-uptake from different models under control conditions. A. Control mice (black); Aging (orange); Diabetes (violet). Pulses of 40 μmol/L free Ca^2+^ were added as indicated by arrow heads. B. Ischemia-reperfusion, IR (green); exposure to 5 μmol/L tert-butyl hydroperoxide, t-BH, (blue). Pulses of 40 μmol/L (red), 0.2 μmol/L (green), and 5 μmol/L (blue) free Ca^2+^ were added respectively as indicated by arrow heads. C-F: Column diagrams of the averaged results under the conditions indicated below. The final concentrations of ADP and CsA are 500 μmol/L and 0.2 μmol/L, respectively. The amount of Ca^2+^ was normalized to the mitochondrial content (mg protein). All values are mean ± SEM. * and ** denote significant difference P < 0.05 and P < 0.01 respectively, between treatment and control within an animal group. ***^#^*** and ^##^ denote significant difference P < 0.05 and P<0.01, respectively, between animal groups with similar treatment.

Figure 2 shows the CRC in the different models of oxidative stress. Ca^2+^ was added consecutively to isolated mitochondria until they reached their maximum uptake capacity, where mPTP opened to release all Ca^2+^, causing an increase in extramitochondrial Ca^2+^ as monitored by Arsenazo III absorbance. Control mitochondria were able to sequester up to 10 pulses of 40 µmol/L free Ca^2+^ with a mean value of 344 ± 32 nmol/mg protein (Figure 1A and Figure 1C). We were surprised to find that chronic oxidative stress did not affect the initial CRC or the effect of ADP (Figure 2C-D). In contrast, acute oxidative stress had an adverse effect on mitochondrial CRC by facilitating mPTP opening. After IR or t-BH exposure, the first addition of 40 µmol/L Ca^2+^ was able to trigger mPTP opening (Figure 2B, red trace). Therefore, 0.2 µmol/L and 5 µmol/L Ca^2+^ additions were used for assessing mitochondrial CRC after IR and t-BH treatments, respectively (Figure 2B). The CRC was dramatically decreased to 1 ± 0.2 nmol/mg protein (IR) and 32 ± 4 nmol/mg protein (t-BH) (Figure 2E-F). 

CsA is a well-known inhibitor of CypD and protects against ischemia-reperfusion damage at a concentration of 0.2 µmol/L [26]. Indeed, this concentration of CsA increased CRC in all models (Figure 2). 500 μmol/L ADP had a similar effect and restored CRC to 447 ± 105 nmol/mg protein after IR and to 514 ± 103 nmol/mg protein after t-BH exposure, respectively (Figure 2E-F). The recovered CRC was not significantly different from that of control mice in the absence of ADP (P = 0.109 for t-BH and P = 0.689 for IR, respectively, Figure 2E-F). Thus, ADP is a potent inhibitor of mPTP – also in mitochondria, which have been exposed to severe oxidative stress.

### Similar effect of ADP irrespective of initial Ca^2+^-uptake capacity

**Figure 3:**
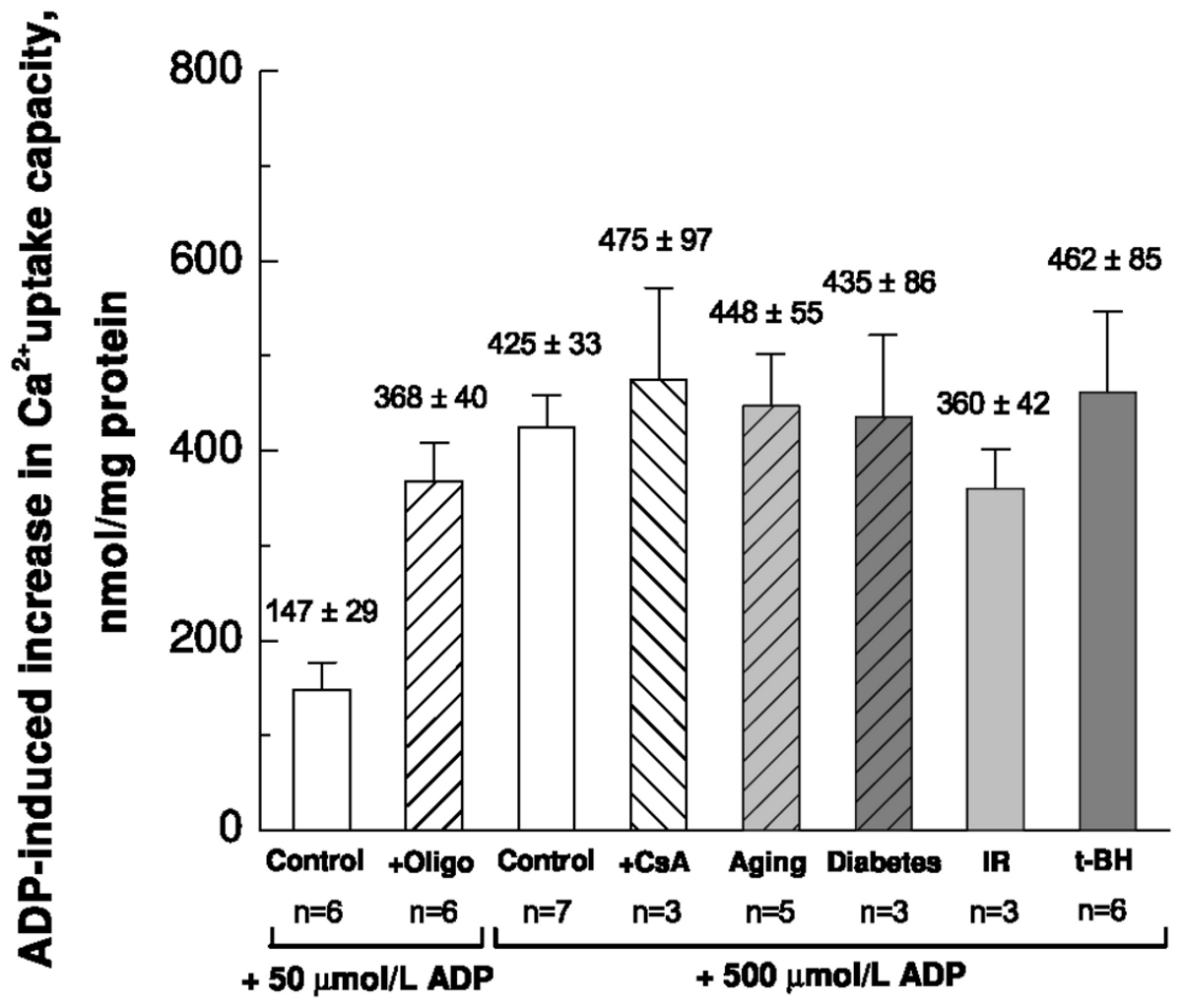
Increase in mitochondrial Ca^2+^-uptake capacity induced by ADP.

Figure 3 is a column diagram showing the effect of ADP – i.e. how much does ADP increase CRC. It illustrates, as noted above, that 50 µmol/L ADP increased CRC much more, when F_1_F_0_-ATPase was inhibited by oligomycin (P = 0.008). But what is in our opinion more remarkable, is the fact that although CRC was drastically lowered by acute oxidative stress (IR and t-BH) to the extent that mitochondrial Ca^2+^-buffering was almost absent (Figure 2E-F), ADP exerted a similar effect as in mitochondria from control mice. In control as well as all model of oxidative stress, 500 µmol/L ADP increased CRC by 360-460 nmol/mg protein (Figure 3).

In mitochondria from control mice, the increase in Ca^2+^-uptake capacity induced by 50 µmol/L ADP in the absence and presence of oligomycin, and 500 µmol/L ADP alone and in the presence of cyclosporine A (CsA) are shown as indicated. In models of chronic and acute oxidative stress, the increase induced by 500 µmol/L ADP is shown as indicated below the columns. The increase induced by 500 µmol/L ADP is remarkably consistent in control and the different models of oxidative stress. 

### ADP attenuates ROS generation in control, but not after exposure to t-BH

**Figure 4:**
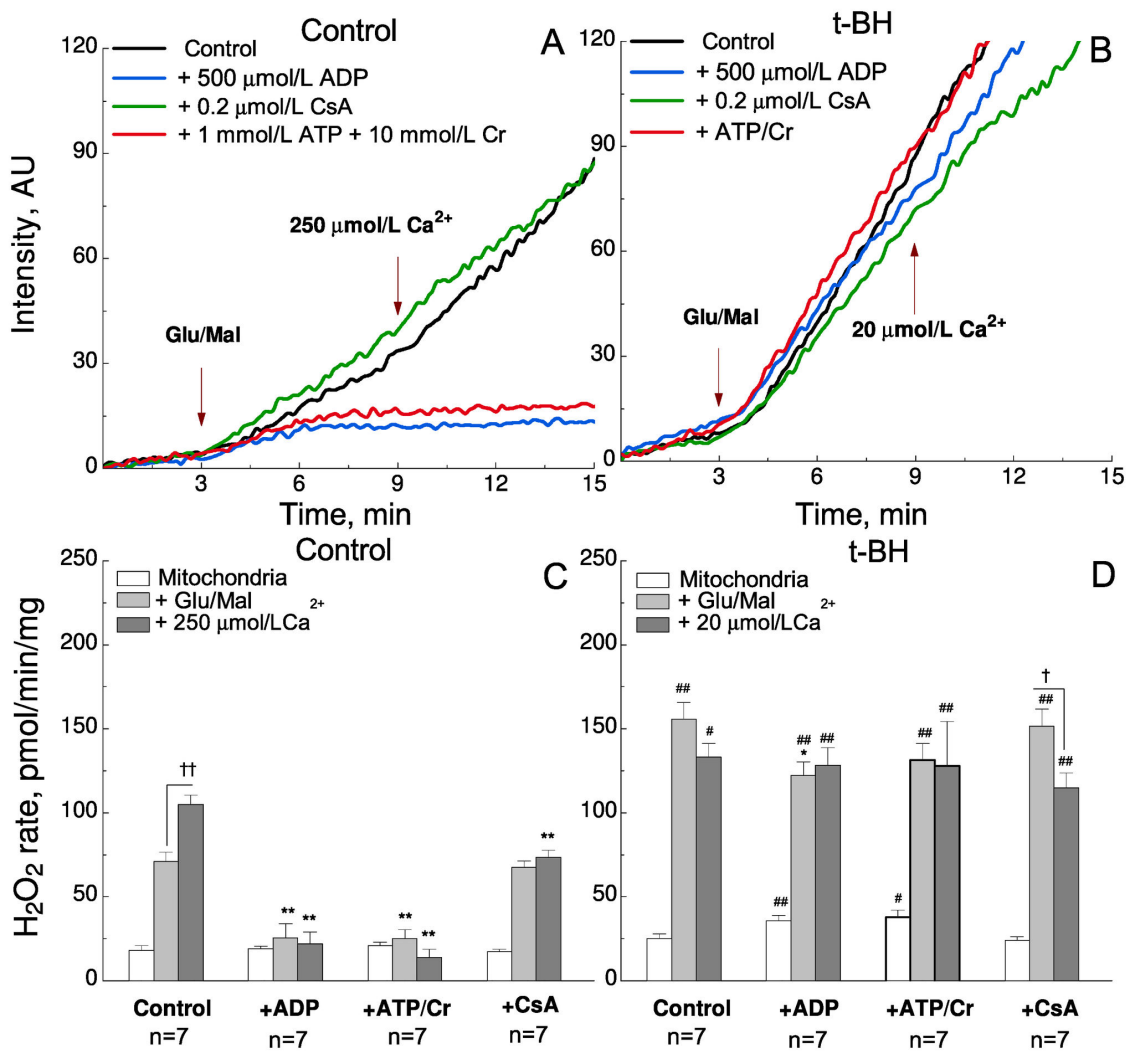
Rate of ROS production in mitochondria from control mice and after t-BH exposure. A-B: Representative raw traces of the ROS-recordings. The rate of hydrogen peroxide (H_2_O_2_) production was followed spectrofluorometrically by its reaction with Amplex Red in the presence of horseradish peroxidase. The rate of ROS production was recorded in three consecutive steps: After addition of mitochondria alone, after addition of substrates (5 mM glutamate and 5 mM malate) and after addition of one submaximal dose of Ca^2+^. Traces are shown under control conditions (no additions; black), in the presence of 500 µmol/L ADP (blue), 0.2 µmol/L CsA (green), and 1mmol/L ATP + 10 mmol/L creatine (Cr) (red). A: control mice; B: after exposure to 5 μmol/L t-BH. C-D: Column diagrams of the averaged results under the conditions indicated below each column. Steady-state H_2_O_2_ production was recorded in the mitochondria alone (rate between 1-3 min; white columns), after addition of substrates (rate between 6.5-8.5 min; light grey columns), and after addition Ca^2+^ (rate between 11-15 min; dark grey columns). Treatments within each group are indicated below the columns. All values are mean ± SEM. † and †† denote significant effect of substrates and/or Ca^2+^, P < 0.05, P < 0.01 respectively. *, ** denote significant difference P < 0.05, and P < 0.01, respectively, between control and different treatments within the group. ***#***and ^##^ denote significant difference (P < 0.05 and P<0.01, respectively) between control and t-BH with the same treatment. The number of experiments is indicated below.

In addition to Ca^2+^, ROS are also potent activators of mPTP. To determine whether ADP-mediated ROS decrease contributes to the inhibition of mPTP, we measured ROS production in the preparations. Amplex Red was used to record ROS production with three consecutive steps: 1) mitochondria alone, 2) after addition of substrates (glutamate and malate), and 3) after the addition of one pulse of a submaximal Ca^2+^-concentration to trigger partial opening of mPTP. The data on ROS production in old and diabetic mice and after IR did not give any new information. Therefore, we show only the results from control and after t-BH exposure. Figure 4A shows original traces of recordings from control mice and the results are summarized in Figure 4C. As expected, ROS production increased upon addition of substrates, as indicated by the increase in the slope of Amplex Red fluorescence trace. Addition of 250 μmol/L Ca^2+^ increased ROS production further (Figure 4A and C, control). ADP or ATP and creatine, which stimulate F_1_F_0_-ATPase to utilize the proton gradient, both significantly abolished the substrate- and Ca^2+^-induced increase in ROS generation (Figure 4C). CsA abolished mostly the Ca^2+^-induced increase in ROS generation, but the net effects were significantly smaller than those of ADP (Figure 4C, P = 0.003 and P = 0.002 for substrate- and Ca^2+^-induced increase in ROS generation, respectively).

In mitochondria exposed to t-BH, ROS-production was much higher than in control (Figure 4B and D). Neither Ca^2+^ nor ADP and CsA had a profound effect on ROS-production. This implies that the ADP-induced increase in mitochondrial Ca^2+^-uptake capacity is not related to a decrease in ROS-production in mitochondria treated with t-BH.

## Discussion

The most notable results from this study are that 1) ADP increases CRC after IR and t-BH exposure to an extent that is remarkably similar to that in control, and 2) the effect of ADP on CRC in mitochondria exposed to t-BH is not the result of a decreased ROS-production. 

### Inhibition of mPTP relates to the concentration of ADP

The main mechanism through which ADP decreases mitochondrial Ca^2+^-sensitivity has been attributed to the binding of ADP to ANT, which stabilizes the transporter in the m-state and prevents pore opening [27]. It was demonstrated that ANT is not a pore-forming component of the mPTP, as mPTP opening is still triggered in mice lacking ANT1 and ANT2 [28]. However, the regulating role of ANT cannot be disputed, as inhibition of ANT in different conformations by bongkrekic acid or atractyloside has opposite effects on mitochondrial Ca^2+^-sensitivity [4]. Haworth and Hunter proposed that ADP exerts its effect by binding to an internal site as well as to ANT [23]. Recent studies suggest that either the mitochondrial phosphate carrier or F_1_F_0_-ATPase could be one of the key components for mPTP [13,14,29,30]. Both cases include a role for ADP [13,29]. In the latter case, ADP affects mPTP via direct binding to F_0_F_1_-ATPase. This, however, does not exclude an additional effect via ADP-binding to ANT. 

The present study showed that the effect of ADP on CRC relates to its concentration. 500 μmol/L ADP had a signifcantly larger effect than 50 μmol/L ADP (Figure 1). Also, 50 μmol/L ADP had a significantly larger effect in the presence of oligomycin to inhibit ADP-turnover by F_1_F_0_-ATPase (Figure 1B-C). Thus, the effect of ADP is specific to [ADP] and not the total concentration of adenine nucleotides.

It is noticed that oligomycin on its own has a negative effect on mitochondrial Ca^2+^-uptake capacity (Figure 1B-C). The mitochondrial membrane potential decreases as the mitochondria take up a significant amout of Ca^2+^. It is conceivable that in the absence of oligomycin, the mitochondria will hydrolyze ATP to ADP in order to maintain their membrane potential. However, oligomycin renders the mitochondria unable to rescue their membrane potential, so that the critical potential at which mPTP opens is reached at a lower Ca^2+^-concentration. 

### Ca^2+^-sequestration has no significant effect on CRC

ADP can buffer Ca^2+^ by itself [31], and could have an additional effect on mitochondrial Ca^2+^ buffering such as the maintenance of high matrix Pi concentrations. It has been shown that ATP-Mg^2-^/Pi^2-^ and HADP^2-^/Pi^2-^ can increase matrix Ca^2+^ buffering via Ca^2+^-Pi precipitates and thus desensitize the mPTP [32,33]. Mitochondrial Ca^2+^-sequestration is proportional to the ADP-uptake [32]. If Ca^2+^-sequestration played a significant role in the effect of ADP, we would expect that the Ca^2+^-uptake capacity is increased more by 500 µmol/L ADP than by 50 µmol/L ADP in the presence of oligomycin. Our results suggest that this is not the case (Figures 1 and 3). However, the role of Ca^2+^-sequestration should be studied further by quantifying intra- and extramitochondrial total and free [Ca^2+^] as mitochondria take up Ca^2+^ after successive Ca^2+^-pulses. 

### Similar effect of ADP on CRC in all models of oxidative stress

Oxidative stress has an unfavourable effect on mitochondrial CRC [5]. We studied the effect of ADP on CRC in different models of increased oxidative stress. Chronic oxidative stress by aging and diabetes had no significant impact on CRC or the effect of ADP. However, the acute oxidative damage caused by IR and t-BH drastically lowered CRC to the extent that mitochondrial Ca^2+^-uptake was almost absent. It is interesting that ADP was able to restore this function (Figures 2E-F) and with an effect that was similar to that in control (Figure 3). It appears that irrespective of the healthiness of mitochondria, a certain amount of ADP increases CRC by a certain amount. This is interesting, because ADP-binding to ANT is reduced by oxidative stress [15]. Therefore, our results suggest that although ADP to some extent inhibits mPTP opening via binding to ANT, significant inhibition also occurs by some other mechanism. 

### ADP does not increase CRC via an effect on ROS-production in mitochondria exposed to t-BH

In addition to Ca^2+^, ROS also effectively trigger mPTP openings. One of the aims of this study was to see whether ADP might affect mitochondrial CRC via ROS decrease in the models of oxidative stress. In our study, we used Amplex red and horseradish peroxidase as a H_2_O_2_ detection system. This allowed us to use the efflux of H_2_O_2_ from mitochondria as a measure of net superoxide production in the matrix. Under physiological conditions, the production of ROS and their consumption in the form of H_2_O_2_ is finely tuned. ROS are mainly produced as superoxide by complexes I and III in the mitochondrial electron transport chain [34]. Superoxide is dismutated to H_2_O_2_ by manganese superoxide dismutase (MnSOD) in the mitochondrial matrix and copper/zinc superoxide dismutase (Cu/ZnSOD) in the mitochondrial intermembrane space and cytosol. The resulting H_2_O_2_ can easily diffuse across membranes to be detected by the assay. However, H_2_O_2_ can also be consumed by matrix enzymes, e.g. glutathione peroxidases, and cause an underestimate of superoxide production by competing with the efflux of H_2_O_2_ [35]. By a series of redox reactions, H_2_O_2_ is converted to H_2_O, while glutathione (GSH) is oxidized to glutathione disulfide (GSSG). GSSG is reduced back to GSH by the oxidation of pyridine nucleotides (NADH/NADPH). The GSH/GSSG ratio provides an estimate of cellular redox buffering capacity, thus playing a key role in maintaining redox homeostasis [36]. 

ADP can regulate mitochondrial ROS production through complexes I and III, as well as general redox state of the mitochondrial matrix (NADH/NADPH, GSH) via oxidative phosphorylation. Indeed, the addition of ADP caused a decrease in H_2_O_2_ export from control mitochondria (Figure 4A and C). However, in t-BH treated mitochondria the fine tuning of ROS production and consumption by ADP in mitochondria is disturbed (Figure 4B-D). t-BH treatment induces the oxidation of pyridine nucleotides [37] and GSH [38]. GSSG is not exported from the mitochondria [39] and increased GSSG formation may decrease the activity of mitochondrial redox-sensitive proteins [40–46]. As a result, H_2_O_2_ export is increased in t-BH mitochondria (Figure 4B-D). Although ADP does reduce ROS, it is not able to bring it down to control level (Figure 4D). Thus, the t-BH results demonstrate that ADP regulates mitochondrial CRC not only via ROS reduction, but via some other mechanisms as well. 

## Conclusion

ADP is well known to desensitize the mPTP to intramitochondrial Ca^2+^, so that the mitochondria can take up more Ca^2+^, before opening of mPTP is triggered. In the present study, we found that ADP had the same effect in severely stressed mitochondria as in control. This suggests that ADP, in addition to its regulation via ANT, exerts significant regulation of mPTP via some other mechanism. Although ADP decreases ROS production in control mitochondria, its effect is minor in t-BH-treated mitochondria. Therefore, we conclude that at least in some cases e.g. t-BH treatment, the ADP-induced increase in mitochondrial Ca^2+^-uptake capacity is not related to a reduction in ROS. It is likely that significant regulation by ADP occurs via its internal binding site (reported in [23]), which could be on F_1_F_0_-ATPase [29].

**Figure 5:**
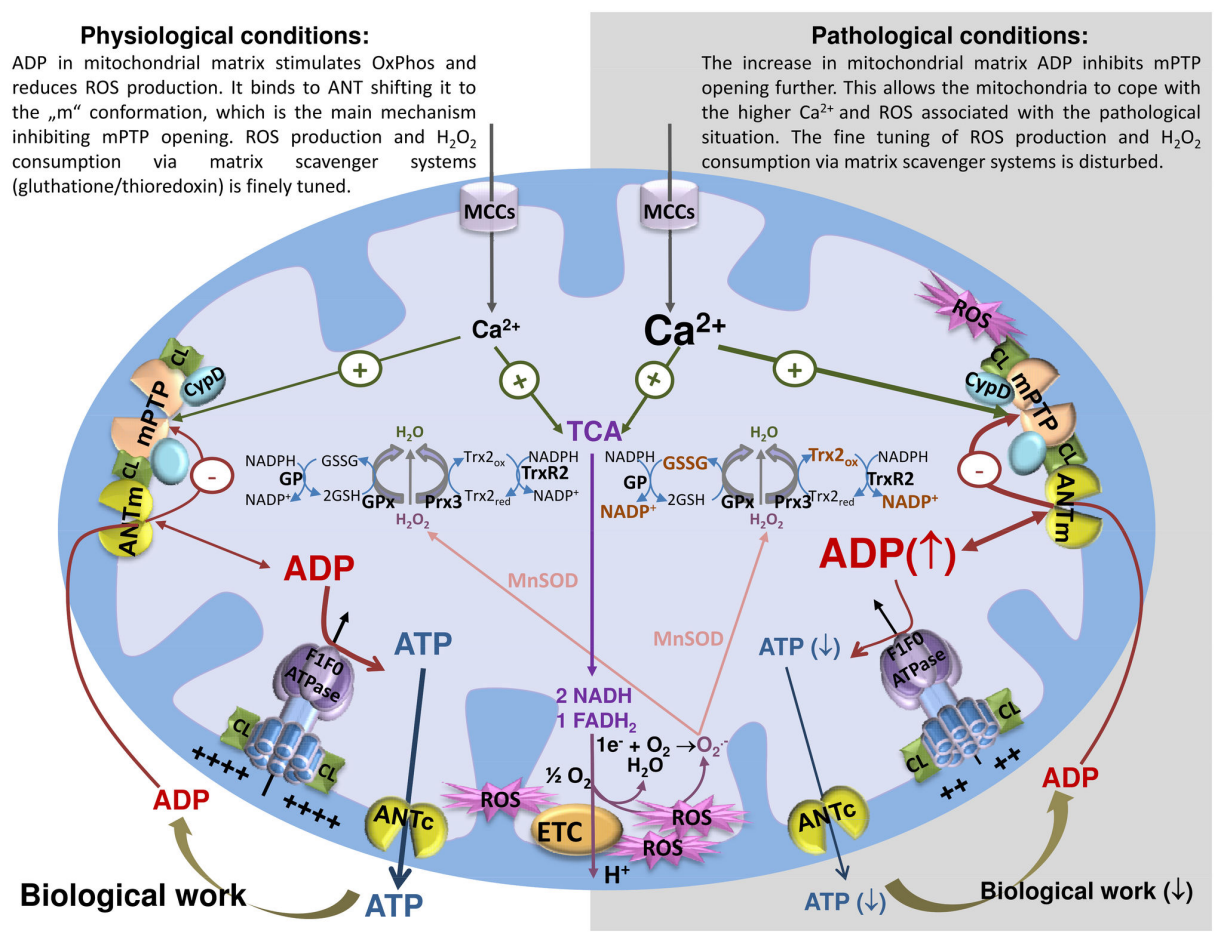
ADP is a master regulator of mitochondrial ATP production, Ca^2+^ homeostasis, and ROS generation. Under physiological conditions, optimal ADP concentrations in mitochondrial matrix serve effectively to maximize ATP generation while minimizing ROS. ROS production via ETC and annihilation via matrix scavenger systems (gluthatione/thioredoxin) is finely balanced. Under pathological conditions, mitochondrial Ca^2+^ and ROS increase, stimulate excessive mPTP opening and thus the signaling pathway for injury. These detrimental effects are countered by a concomitant increase in ADP, which allows the mitochondria to endure a larger load of Ca^2+^ and ROS, maintaining mitochondrial integrity and function when it is most critical. ANTm – ANT in the “m”-conformation; ANTc – ANT in the “c”-conformation; CL - cardiolipin; CypD – cyclophilin D; ETC – electron transport chain; GR – glutathione reductase; GRx – glutathione peroxidase; GSH – reduced glutathione; GSSG – oxidized glutathione; MCCs – mitochondrial calcium channels; MnSOD – manganese superoxide dismutase; mPTP – mitochondrial permeability transition pore; Prx3 – peroxiredoxin 3; ROS – reactive oxygen species, TCA – tricarboxylic acid cycle;  Trx2_red_ - reduced thioredoxin 2; Trx2_ox_ – oxidized thioredoxin 2; TrxR2 – thioredoxin reductase 2.

From a more integrated point of view, it is very interesting that ADP is as potent an inhibitor of mPTP in severely stresssed mitochondria, and that its effect relates to the concentration. Namely, ADP concentrations increase in the diseased state due to less efficienct ATP production, depolarized mitochondrial membrane potential, Ca^2+^ overload, and a smaller pH gradient [47–49]. It is plausible that this increase in ADP concentration could protect against opening of mPTP and thus delay the injury and death of cells. This function of ADP as a regulator of mitochondrial ATP production, Ca^2+^ homeostasis, and ROS generation is shown schematically in Figure 5. Under physiological conditions, it serves as a substrate for ATP synthesis while ensuring the closure of mPTP and modest ROS generation. Under pathological conditions like IR injury, when the mitochondria are most vulnerable for mPTP opening, ADP can effectively delay mPTP opening so that mitochondrial membrane integrity can be preserved to maintain energy production. The ability of ADP to serve multiple roles as a potent mPTP blocker, a substrate for catalysing ATP generation, and an inhibitor for ROS generation, makes it a unique molecule to preserve mitochondrial function under pathophysiological conditions.
